# SARS-CoV-2 variants of concern and spike protein mutational dynamics in a Swedish cohort during 2021, studied by Nanopore sequencing

**DOI:** 10.1186/s12985-022-01896-x

**Published:** 2022-10-18

**Authors:** Steinar Mannsverk, Julia Bergholm, Navaneethan Palanisamy, Patrik Ellström, René Kaden, Johan Lindh, Johan Lennerstrand

**Affiliations:** 1 Department of Medical Sciences, Section for Clinical Microbiology and Hospital Hygiene, Uppsala University, 751 85 Uppsala, Sweden; 2 Department of Cell and Molecular Biology, Uppsala University, 752 37 Uppsala, Sweden; 3 Chester Medical School, University of Chester, CH2 1BR Chester, UK

**Keywords:** SARS-CoV-2, Spike, Mutations, Variants of concern, Delta variant, ARTIC network, Nanopore sequencing

## Abstract

**Background:**

Since the beginning of the COVID-19 pandemic, new variants of significance to public health have emerged. Consequently, early detection of new mutations and variants through whole-genome sequencing remains crucial to assist health officials in employing appropriate public health measures.

**Methods:**

We utilized the ARTIC Network SARS-CoV-2 tiled amplicon approach and Nanopore sequencing to sequence 4,674 COVID-19 positive patient samples from Uppsala County, Sweden, between week 15 and 52 in 2021. Using this data, we mapped the circulating variants of concern (VOC) in the county over time and analysed the Spike (S) protein mutational dynamics in the Delta variant throughout 2021.

**Results:**

The distribution of the SARS-CoV-2 VOC matched the national VOC distribution in Sweden, in 2021. In the S protein of the Delta variant, we detected mutations attributable to variants under monitoring and variants of interest (e.g., E484Q, Q613H, Q677H, A222V and Y145H) and future VOC (e.g., T95I and Y144 deletion, which are signature mutations in the Omicron variant). We also frequently detected some less well-described S protein mutations in our Delta sequences, that might play a role in shaping future emerging variants. These include A262S, Q675K, I850L, Q1201H, V1228L and M1237I. Lastly, we observed that some of the Delta variant’s signature mutations were underrepresented in our study due to artifacts of the used bioinformatics tools, approach and sequencing method. We therefore discuss some pitfalls and considerations when sequencing SARS-CoV-2 genomes.

**Conclusion:**

Our results suggest that genomic surveillance in a small, representative cohort can be used to make predictions about the circulating variants nationally. Moreover, we show that detection of transient mutations in currently circulating variants can give valuable clues to signature mutations of future VOC. Here we suggest six such mutations, that we detected frequently in the Delta variant during 2021. Lastly, we report multiple systematic errors that occurred when following the ARTIC Network SARS-CoV-2 tiled amplicon approach using the V3 primers and Nanopore sequencing, which led to the masking of some of the important signature mutations in the Delta sequences.

**Supplementary Information:**

The online version contains supplementary material available at 10.1186/s12985-022-01896-x.

## Background

Despite strong vaccination campaigns and control measures enforced by most countries around the globe, the COVID-19 pandemic is still not under control. From its discovery in December 2019 until the 7th of April 2022, 495,207,407 cases and 6,167,296 deaths have been confirmed globally (https://coronavirus.jhu.edu, accessed on the 7th of April 2022). Although the virus possesses an RNA-dependent RNA polymerase (RdRp) with proofreading activity, new variants are on the rise at regular intervals, making outbreak control challenging. New variants arise due to the error-prone nature of the viral RdRp, random template switching during RNA replication and genome plasticity [1]. Since the first report of SARS-CoV-2 in China, numerous new variants with altered characteristics have emerged. Consequently, the World Health Organization’s (WHO) Technical Advisory Group on Virus Evolution have classified some of these variants into three main groups: variants under monitoring (VUM), variants of interest (VOI) and variants of concern (VOC) (https://www.who.int/en/activities/tracking-SARS-CoV-2-variants/). VUM encompass variants with genetic changes suspected to affect virus characteristics, while VOI have in addition to this demonstrated significant community transmission in several clusters or multiple countries. VOC, on the other hand, consist of variants that have exhibited enhanced transmissibility or virulence, a negative impact on public health measures and/or resistance to available diagnostics, therapeutics or vaccines. Currently, there are five VOC, each designated with a Greek alphabet letter: Alpha, Beta, Gamma, Delta and Omicron (https://www.who.int/en/activities/tracking-SARS-CoV-2-variants/).

Coronaviruses cause disease in both animals and humans. In humans, the symptoms can range from a mild cold and fever to pneumonia and severe acute respiratory syndrome [2]. Some coronaviruses can jump from the animal reservoir to humans (zoonosis) either directly or via an intermediary host [1, 3, 4]. In the last two decades, we have had three new introductions of zoonotic Coronaviruses that have caused outbreaks in humans. This includes the severe acute respiratory syndrome coronavirus (SARS-CoV or SARS-CoV-1) outbreak between 2002 and 2004, the Middle-East respiratory syndrome-related coronavirus (MERS-CoV) outbreak in 2012 and more recently, the ongoing severe acute respiratory syndrome coronavirus 2 (SARS-CoV-2 or COVID-19) pandemic [5]. For all three of these viruses, detailed information on how they crossed the animal-human barrier is still lacking [4, 6]. At the nucleotide level, SARS-CoV-2 shows high sequence similarity with SARS-CoV-1 (86.85%) and MERS-CoV (81.25%) [7]. SARS-CoV-2 is spread via respiratory droplets, aerosols and fomites. In the human lungs, the virus attaches to the cell membrane-bound angiotensin-converting enzyme 2 (ACE2) via the Spike (S) protein [8]. With the help of the host serine protease TMPRSS2, the viral membrane fuses with the host cell membrane, thereby delivering the viral genome into the cell [8]. Inside the cell, the virus utilizes the host’s resources for its replication. The observed pathology due to COVID-19 mainly stems from the excessive destruction of lung tissue.

Due to the central role of the S protein in the viral life cycle, vaccines and monoclonal antibodies (mAbs) targeting this protein have been developed and approved for human use, to stop the spread of the virus [9, 10]. Multiple studies have shown that some of the mutations accumulated in the viral S protein can confer resistance towards vaccine-induced antibodies, as well as mAbs and convalescent plasma [11–22]. The global prevalence of such mutations, especially in the receptor-binding motif, is high [23]. Therefore, it becomes of utmost importance to detect new mutations and variants circulating in the community early, to employ appropriate public health measures in time.

Uppsala County has 395,026 inhabitants (as of December 2021) and Uppsala, the capital of the county, is the fourth largest city in Sweden (https://www.scb.se/en/finding-statistics/statistics-by-subject-area/population/population-composition/population-statistics/pong/tables-and-graphs/quarterly-population-statistics--municipalities-counties-and-the-whole-country/quarter-4-2021/, accessed on the 1st of April 2022). In the municipality of Uppsala, where most of the population resides, more than one-third of the residents have a foreign background or citizenship, with many being non-European (https://www.uppsala.se/kommun-och-politik/publikationer/2014/befolkningsstatistik, accessed on the 1st of April 2022). Further, commuting between Uppsala and Stockholm (the capital and the most populated city in Sweden) is common. Therefore, within the county there is a continuous influx of people from other parts of Sweden and abroad, promoting the swift appearance and spread of new SARS-CoV-2 variants circulating globally. In the present study, we sequenced COVID-19 positive patient samples collected in Uppsala County between week 15 and 52 in 2021, using the ARTIC Network SARS-CoV-2 tiled amplicon approach and Nanopore sequencing [24]. From the obtained data, we studied the circulating VOC and S protein mutations in the Delta variant over time, within the county. Furthermore, from sequencing the Delta variant, we observed that some of this variant’s signature mutations were underrepresented. We therefore discuss some pitfalls and considerations when employing the bioinformatics tools, ARTIC Network approach and Nanopore sequencing method.

## Methods

### Sample origin and viral RNA extraction

In Uppsala County, Sweden, all residents experiencing symptoms related to COVID-19 were recommended to get tested for SARS-CoV-2, as part of the national guidelines during the COVID-19 pandemic. Nasopharyngeal/oropharyngeal or saliva samples collected from the residents were analysed at the Section for Clinical Microbiology and Hospital Hygiene, Uppsala University Hospital, Sweden. Only SARS-CoV-2 positive samples with a Ct value < 31 (from the hospital’s in-house quantitative reverse transcription PCR (RT-qPCR) test) were selected for whole-genome sequencing. This was because positive samples above this threshold generally contained too little viral RNA for sequencing. Between week 26 and 47, all SARS-CoV-2 positive samples with a Ct value < 31 were sequenced. For the remaining weeks, representative/targeted sampling of samples with a Ct value < 31 was employed, as recommended by the European Centre for Disease Prevention and Control (ECDC) (https://www.ecdc.europa.eu/en/publications-data/guidance-representative-and-targeted-genomic-sars-cov-2-monitoring). Viral RNA was extracted from the samples using a Chemagic™ 360 (PerkinElmer, USA) or an easyMAG® (bioMérieux, France) instrument, according to the respective manufacturer’s instructions. The study was approved by the Swedish Ethical Review Authority (Etikprövningsmyndigheten) under the case number dnr 2022-01249-01 and carried out according to the ethical standards of Uppsala university hospital.

### SARS-CoV-2 library preparation and sequencing

The ARTIC Network SARS-CoV-2 tiled amplicon approach was followed for the amplicon generation and library preparation of the SARS-CoV-2 genome isolated from the patients [24]. For the sequencing, we followed the protocol from Oxford Nanopore Technologies® (version PTCN_9103_v109_revG_13Jul2020) with slight modifications. The reverse transcription PCR was done using the NEBNext® ARTIC SARS-CoV-2 Companion kit (Oxford Nanopore Technologies®, UK) from New England Biolabs, USA, with the ARTIC Network SARS-CoV-2 V3 primers. Next, the sequencing library was prepared using the Native Barcoding Expansion 96 (EXP-NBD196) and Ligation Sequencing (SQK-LSK109) kits both from Oxford Nanopore Technologies®, UK. The library concentration was measured using the Qubit HS dsDNA assay kit (Thermo Fisher, USA). The sequencing was carried out on the GridION with the R9.4.1 flow cells (Oxford Nanopore Technologies®, UK). The MinKNOW software (version 21.10.8) was used for operating the device and “high-accuracy” base-calling was selected. The barcode settings were set as follows: barcodes on both ends, a minimum barcoding alignment score of 60 and a mid-read barcoding alignment score of 50. Additionally, the barcodes were trimmed off after base-calling.

### Bioinformatic analysis

The fastq sequences from the GridION were assembled and analysed in Geneious Prime (version 2021.1.1). Firstly, the fastq files were trimmed using the Geneious plugin BBDuk (version 38.84) (https://sourceforge.net/projects/bbmap/). All 196 primer sequences of the ARTIC Network SARS-CoV-2 V3 primers were trimmed from the left end with a Kmer length of 21 and a maximum of three substitutions. Low-quality ends (Q < 10) were trimmed (both ends) and reads < 50 nucleotides were discarded. Next, the trimmed reads were aligned to the SARS-CoV-2 isolate Wuhan-Hu-1 (NCBI accession: NC_045512.2). This was done using the Minimap2 plugin (version 2.17), with the data type “Oxford Nanopore’’ selected and secondary alignments set to their default values [25]. A consensus sequence was generated from the aligned reads using Geneious’ built-in “Generate Consensus Sequence” tool. A minimum coverage of four reads and a minimum nucleotide frequency of 0.5 were applied to call a base. “Trim to reference sequence” was also enabled. The consensus sequences were consequently deposited into the Global Initiative on Sharing Avian Influenza Data (GISAID) database (https://www.gisaid.org/). Only fasta sequences with < 5000 ambiguities were uploaded and sequences with early frameshifts and consequently premature stop codons in the S protein were omitted. The submission ID of all deposited sequences included in this study is provided in the Supplementary Materials.

For the S protein mutational analysis in the Delta variant, the trimmed reads were mapped to the S gene of the SARS-CoV-2 isolate Wuhan-Hu-1 using the same Minimap2 settings. Mutations in the S protein were analysed using the “Find Variations/SNPs” tool in Geneious. Minimum coverage and variant frequency were set to 10 and 0.70, respectively. The maximum variant p-value was set to 10^− 6^. The mutations were applied to the reference sequence using the “Apply Variants to Reference Sequence” tool in Geneious with all options left unchecked, resulting in fasta sequences covering the S gene.

### Data science using Python

Metadata of all the SARS-CoV-2 sequences from Uppsala County deposited to the GISAID database was downloaded by specifying “hCoV-19/Sweden/UUH” in the virus name search option of EpiCoV. All search results were selected and downloaded in the “Augur Pipeline” format. The resulting .csv file was loaded into Python (version: 2.7.10; https://www.python.org) and organized using the Pandas package [26]. Each sample was then organized by sampling week and VOC, according to the pangolin lineage system (https://cov-lineages.org). Plots were generated using the Matplotlib Pyplot package in Python [27].

For the S protein mutational analysis in the Delta variant, the generated fasta sequences covering the S protein of our Delta sequences were used. Since the Delta variant has nine signature mutations in its S protein and we could not detect at least two of these, we only included fasta sequences containing a minimum of seven single nucleotide polymorphisms compared to the reference sequence (https://gvn.org/covid-19/delta-b-1-617-2/). The rationale was to omit sequences with low coverage regions from our mutational analysis. Fasta sequences meeting this quality criterion were uploaded to the online COVID-19 genome annotator tool [28]. The resulting .csv file containing all detected amino acid mutations in the S protein was loaded into Python and organized using the Pandas package. Only samples with the amino acid mutations L452R, T478K and P681R in the S protein (signature mutations in the Delta variant) were retained. Another dataset containing the sampling week matched with the sample ID for each sequence was also loaded in, to organize the amino acid mutations by sampling week. The S protein mutation heatmap was generated using the Seaborn package (version: 0.11.2) in Python (https://github.com/mwaskom/seaborn/tree/v0.8.1).

## Results

### Mapping the circulating SARS-CoV-2 VOC in Uppsala county in 2021

**Fig. 1:**
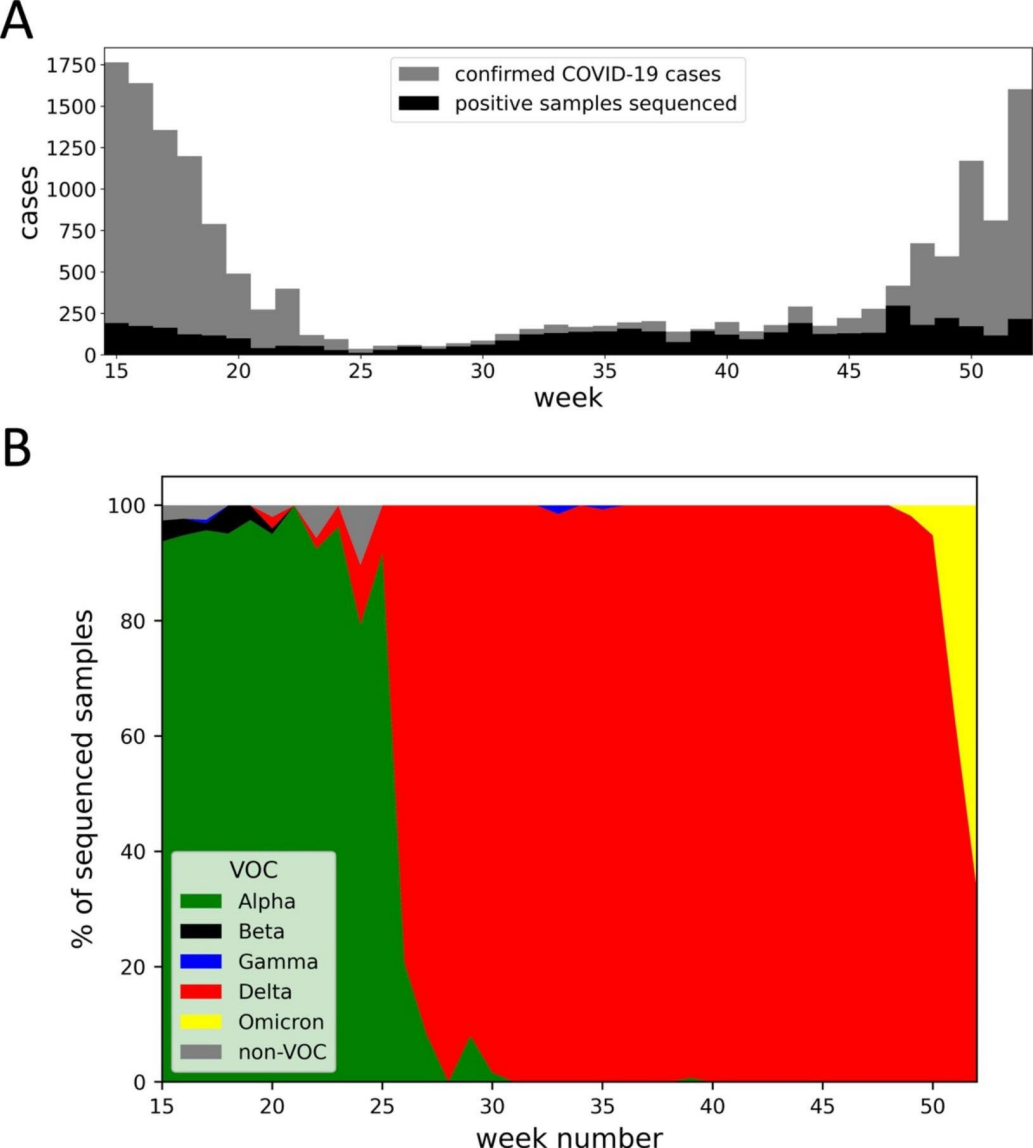
Circulating SARS-CoV-2 variants of concern (VOC) in Uppsala County, Sweden during 2021. (A) Shows the week-wise distribution of the number of confirmed COVID-19 cases in Uppsala County, Sweden (grey) and the number of positive samples that were subsequently sequenced (black). The number of confirmed cases (per week) was collected from the Swedish Public Health Agency’s official COVID-19 statistics (https://www.folkhalsomyndigheten.se/smittskydd-beredskap/utbrott/aktuella-utbrott/covid-19/statistik-och-analyser/bekraftade-fall-i-sverige/, accessed on the 25th of March 2022). (B) Shows the distribution of the SARS-CoV-2 VOC (or non-VOC) during 2021 (between week 15 and 52) from the COVID-19 positive samples that were sequenced in our laboratory. The distribution is displayed as percentage (%) of sequenced samples per week. VOC classifications: according to the pangolin lineage system and WHO’s definition of VOC (https://cov-lineages.org; https://www.who.int/en/activities/tracking-SARS-CoV-2-variants/).

As part of the Swedish national genomic surveillance effort during the COVID-19 pandemic, in 2021, a total of 4,674 SARS-CoV-2 sequences from COVID-19 positive patients in Uppsala County were sequenced and submitted to the GISAID database. Between week 26 and 47, we sequenced over 50% of all samples in Uppsala County testing positive for SARS-CoV-2 by RT-qPCR, giving us representative data to map the circulating variants (Fig. 1A). For the remaining weeks, a minimum of 10% of all positive COVID-19 samples each week were sequenced. Sequences submitted by us to the GISAID database were used to determine VOC that were circulating in the county at a particular time in 2021. The Alpha variant was found to be the dominant variant circulating from week 15 to 25 (Fig. 1B). From week 26 to 51, the Delta variant was the dominant variant. While in week 52, the Omicron variant accounted for over 50% of all positive samples sequenced. The prevalence of the Beta and Gamma variants was low in Uppsala County between week 15 and 52 (Fig. 1B). When Delta was the dominant variant circulating in Uppsala County (between week 26 and 51), the median positive COVID-19 cases per week was 178 (45 per 100,000 inhabitants) (Fig. 1A). Interestingly, the distribution of different VOC circulating during 2021 corresponded well between Uppsala County and the rest of Sweden (Fig. 1B; https://www.folkhalsomyndigheten.se/smittskydd-beredskap/utbrott/aktuella-utbrott/covid-19/statistik-och-analyser/sars-cov-2-virusvarianter-av-sarskild-betydelse/, accessed on the 26th of March 2022). This suggests that genomic surveillance at the local level can be a valuable tool to make predictions about the circulating variants nationally.

### S protein mutational dynamics in the Delta variant

Since we sequenced a high proportion of positive COVID-19 samples during the weeks where the Delta variant was the dominant variant circulating in Uppsala County (Fig. 1A and B), we decided to analyse the mutational dynamics of the S protein in the Delta variant in more detail. Out of 3,284 S protein sequences classified as the Delta variant, 1,053 (32%) sequences met our quality criteria and were consequently analysed for mutations in the S protein (see Materials and Methods: Data science in Python for more details). In total, we detected 63 different amino acid substitutions/deletions in the S protein of our Delta sequences (excluding the signature mutations for the Delta variant). However, from week 26 to 51 we did not observe a general increase in the mutational frequency in the S protein (e.g., number of mutations per S protein sequence). For example, in July and December 2021 we detected on an average 5.33 and 5.35 mutations in the S protein per Delta sequence, respectively. This suggests that the S protein mutational dynamics of the Delta variant did not increase significantly throughout the year in Uppsala County. Of note, the Delta variant has nine S protein signature mutations compared to the Wuhan-Hu-1 strain, which is higher than what we detected in the S protein of our Delta sequences (https://gvn.org/covid-19/delta-b-1-617-2/).

**Fig. 2:**
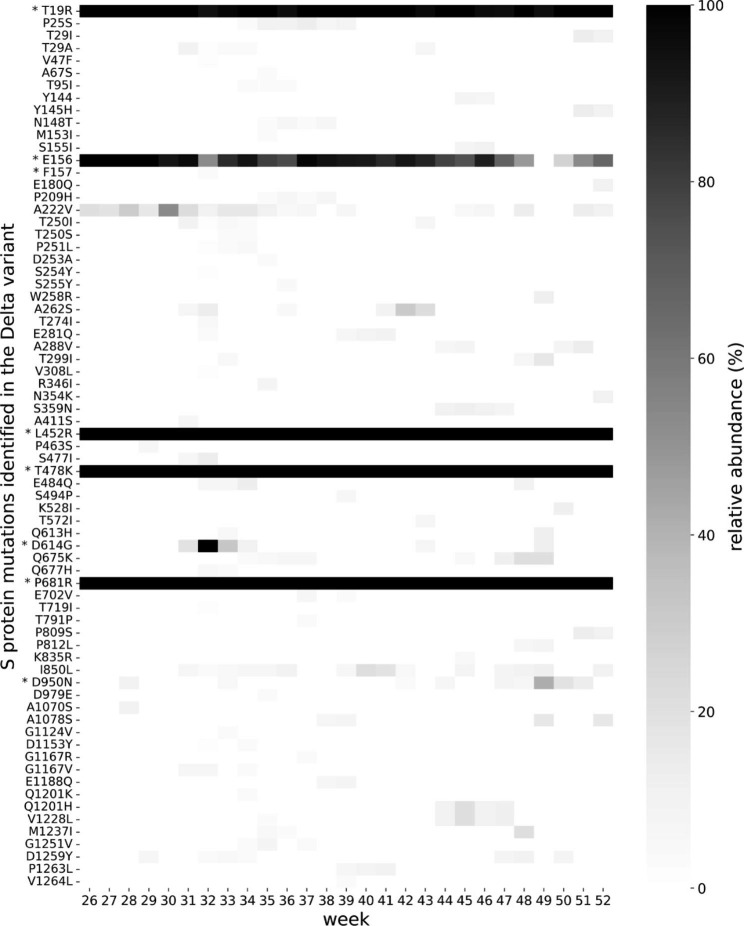
Spike (S) protein mutations detected in the Delta variant circulating in Uppsala County during 2021. The heatmap displays the relative abundance of different S protein amino acid substitutions/deletions (per week) from all the analysed Delta sequences (n = 1053). The intensity scale on the right indicates the percentage (%) of the sequences exhibiting that mutation (white = 0%; black = 100%). Y-axis shows the amino acid substitutions/deletions with the residue number corresponding to the S protein of the SARS-CoV-2 Wuhan-Hu-1 isolate (NCBI accession: NC_045512.2). The character before and after the residue number signify the wild type and substituted amino acids, respectively. The absence of a character after the residue number indicates an amino acid deletion. To reduce random sequencing errors, mutations detected in only one sequence during a week were omitted from that week. Signature mutations for the Delta variant are marked with an asterisk (*). R158G is not shown because it was not detected (https://gvn.org/covid-19/delta-b-1-617-2/ ).

Further, in our S protein mutational analysis of the Delta sequences, we detected multiple mutations assigned as VUM or VOI by the ECDC. Delta + E484Q (That is, a Delta variant having an E484Q substitution in the S protein) was assigned as a VUM by the ECDC in week 34 (https://www.ecdc.europa.eu/sites/default/files/documents/Variants-page-changelog-21-10.pdf, accessed on the 26th of March 2022). We detected this substitution in multiple sequences between week 32 and 34, and 48, in our Delta sequences (Fig. 2). This variant was introduced into Uppsala County twice independently, by travellers returning from southern Europe [29]. We also detected the mutations Q613H and Q677H, separately, several times in the S protein of our Delta sequences, which were both defined as VUM by the ECDC during 2021 (Fig. 2; https://www.ecdc.europa.eu/sites/default/files/documents/Variants-page-changelog-21-10.pdf, accessed on the 26th of March 2022). In week 42, the ECDC assigned the Delta pangolin sub-lineage AY.4.2 as a VOI (https://www.ecdc.europa.eu/sites/default/files/documents/Variants-page-changelog-21-10.pdf, accessed on the 26th of March 2022), carrying the additional S protein mutations Y145H and A222V. We also detected these mutations together in the S protein of our Delta sequences (i.e., we detected sequences classified as AY.4.2) (Fig. 2).

Since December 2021, the Omicron variant was the dominant SARS-CoV-2 variant circulating globally (https://www.gisaid.org/). The variant was first detected in South Africa and reported to the WHO in week 47 [30]. Interestingly, we detected two Omicron signature mutations in our Delta sequences, before the appearance of the Omicron variant. T95I was detected already in week 34 while Y144 deletion was detected in week 45 and 46 (Fig. 2; https://www.ecdc.europa.eu/en/covid-19/variants-concern). Additionally, by comparing the residue positions of the S protein of our Delta sequences with that of the Omicron variant, we detected four amino acid substitutions at the same residue positions with a different amino acid. This included A67S in week 35, Y145H in week 51 and 52, S477I in week 31 and 32 and, as discussed above, E484Q was detected in week 32 to 34, and 48 (Fig. 2; https://www.ecdc.europa.eu/en/covid-19/variants-concern).

Mutations located in the receptor-binding domain (RBD) of the S protein are of particular interest in the context of SARS-CoV-2 evolution because they can confer resistance towards vaccine-induced antibodies, as well as mAbs and convalescent plasma [11–22]. In the S protein RBD of our Delta sequences, we detected the mutations R346I, S477I, E484Q and S494P (Fig. 2). E484Q and S494P have been identified as adaptive mutations in the RBD and were among some of the most frequent mutations observed in the SARS-CoV-2 sequences globally [23, 31]. We also detected several less well-described S protein mutations in the Delta variant circulating in Uppsala County during 2021, which could emerge as signature mutations in future VOC (Table 1). Searching all the sequences deposited to the GISAID database globally reveals that these mutations have been detected in other sequences as well, although some more frequently than others. Interestingly, some of these mutations are also cited in other research articles, but they were not studied in detail (Table 1). In fact, most of the articles only cited the mutation in a large table of detected mutations without any further description.

**Table 1:** Less well-described mutations detected in the S protein of the Delta variant in Uppsala County during 2021. The listed mutations were detected over several weeks. From all the Delta sequences analysed, the listed mutations had a relative abundance of above 20% for at least one week in 2021

S protein amino acid substitution	Number of times detected	Number of sequences in GISAID carrying the mutation^1^	Publications quoting the mutation in PubMed Central (mutation cited in the abstract)^2^
A262S	50	26,810	33 (1)
Q675K	32	1,785	4 (0)
I850L	66	38,719	0 (0)
Q1201H	19	921	0 (0)
V1228L	20	15,993	8 (0)
M1237I	15	14,664	11 (0)

1 = A search was conducted in the GISAID database for all sequences containing the corresponding substitution (https://www.epicov.org/, accessed on the 7th of April 2022). 2 = A search was conducted in the PubMed Central database for articles from 2019 until today, containing the keywords “SARS-CoV-2” and the corresponding mutation. Next, the search was repeated but the keywords had to be present in the abstract to yield a result (https://www.ncbi.nlm.nih.gov/pmc/, accessed on the 7th of April 2022)

### Multiple Delta variant signature mutations were underrepresented in our Delta sequences

When analysing the S protein of our Delta sequences, we observed that multiple Delta variant signature mutations were underrepresented. In particular, the mutations F157, D614G and D950N were only detected in a few sequences, while R158G was never detected (Fig. 2). As mentioned above, the Delta variant has nine signature mutations in the S protein, but we only detected 5.46 mutations on average, per S protein sequence. This can be explained by the lack of these four Delta variant signature mutations in our Delta sequences. We investigated whether this is a unique error in our dataset or whether other laboratories sequencing SARS-CoV-2 genomes globally also experienced this error. For this, we used the Outbreak.info database which displays the relative abundance of different mutations in the S protein of all the Delta sequences deposited to the GISAID database globally. Interestingly, we found that the E156/F157/R158G and D950N mutations were not detected in ~ 8% and ~ 5%, respectively, of all the Delta sequences deposited to the GISAID database globally (https://outbreak.info/compare-lineages, accessed on the 26th of March 2022). However, the D614G mutation was detected in > 99% of the Delta sequences deposited globally.

**Fig. 3:**
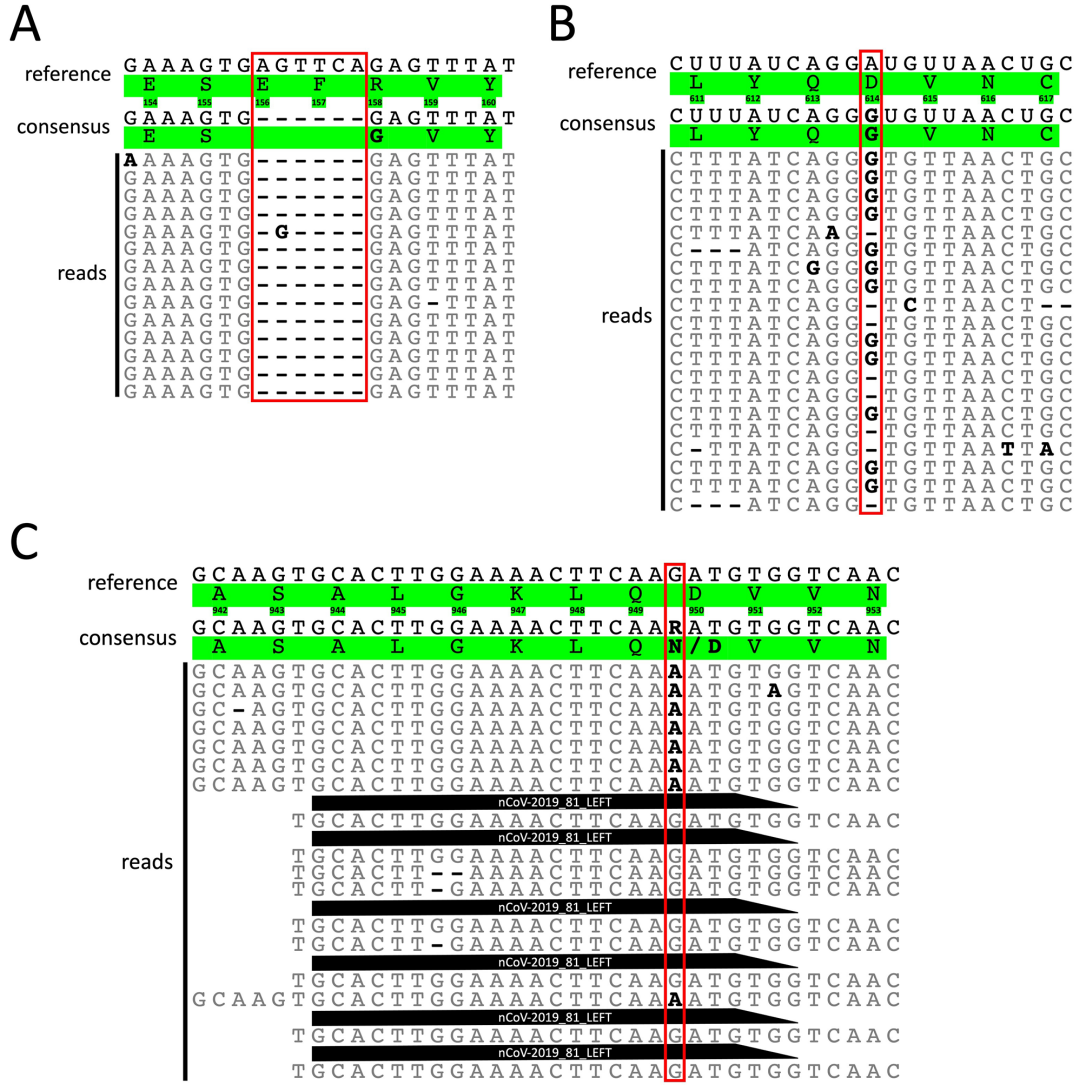
Masking of the Delta variant’s signature mutations due to the bioinformatics tools used or due to systematic sequencing errors. (A), (B) and (C) shows reads aligned to the S protein of the SARS-CoV-2 Wuhan-Hu-1 isolate (NCBI accession: NC_045512.2) from three representative COVID-19 positive samples. The first and second rows above the reads show the reference and consensus sequences (from the read assembly), respectively. White and green backgrounds represent nucleotide and amino acid sequences, respectively. The number below the reference amino acid sequence represents the residue position in the S protein of the SARS-CoV-2 Wuhan-Hu-1 isolate (reference sequence). Reads aligned to the reference sequence are represented below the consensus sequence where bold black characters represent nucleotides differing from the reference sequence. Gaps are represented by dashes (-). (A) Shows residue positions 154 to 160. A non-codon-aligned deletion of six nucleotides is expected between 156 and 158, leading to the triple mutation E156/F157/R158G in the Delta sequences. All reads have the expected six nucleotide deletions (red rectangle), therefore the sequencing and read assembly were correct. (B) Shows residue positions 611 to 617. An A to G nucleotide substitution is expected at residue position 614, leading to the amino acid substitution D614G. Multiple reads exhibit a gap at this position, thereby lowering the frequency of reads exhibiting the nucleotide G at this position (red rectangle). (C) Shows residue positions 942 to 953. The G to A nucleotide substitution is expected at residue position 950, leading to the amino acid substitution D950N. Many reads exhibit the ARTIC Network SARS-CoV-2 V3 primer sequence at its 5’ end (black trapezoid, primer name in white) likely as a result of inefficient primer trimming when using Geneious. Consequently, primer sequences are introduced in the read alignment, thereby lowering the frequency of reads exhibiting nucleotide A (red rectangle). In this case, it masks the D950N amino acid substitution.

The Delta variant’s signature triple mutation E156/F157/R158G is a result of a non-codon-aligned deletion of six nucleotides in the S protein. However, in our mutation heatmap, we only detected the E156 deletion (and the F157 deletion in a few sequences). A closer investigation of the read alignment around this region revealed that our consensus sequences correctly carry the expected six nucleotide deletions (Fig. 3A). However, the COVID-19 genome annotator tool utilized to detect amino acid mutations from our consensus nucleotide sequences was unable to discern this deletion of six nucleotides, hence only the E156 deletion was detected. Consequently, these Delta signature mutations did not appear in our S protein mutation heatmap (Fig. 2), even if they were present in our consensus nucleotide sequences.

Investigation of the aligned reads around residue position 614 revealed that a frameshift deletion occurred in some reads at this position (Fig. 3B). This resulted in a lower frequency of reads containing the nucleotide leading to the amino acid substitution D614G. We evaluated the average frequency of a G nucleotide at this position (leading to the amino acid substitution D614G) in over 1000 of our sequences defined as Delta and found it to be 0.61 ± 0.11 (mean ± standard deviation) in each sequence alignment. Since the minimum variant frequency to call a base was set to 0.70, it explains why D614G was rarely detected in our S protein mutational analysis (see Materials and Methods: Bioinformatic analysis for more details). However, between week 31 and 34, three sequencing runs were executed with suboptimal barcoding score settings in the MinKNOW software, resulting in the appearance of the D614G mutation (Fig. 2). The minimum barcoding score for these runs was set to 40 while matching barcodes at both ends and mid-read barcodes were disabled. To determine whether this was caused by the ARTIC Network SARS-CoV-2 tiled amplicon approach or Nanopore sequencing, we sequenced 78 samples by Nanopore sequencing after preparing them separately both by the ARTIC Network and Midnight approaches [24, 32]. The average frequency of the G nucleotide in the reads was 0.52 ± 0.06 (mean ± standard deviation) when following the ARTIC Network approach and 0.85 ± 0.03 (mean ± standard deviation) when following the Midnight approach. This suggests that the single nucleotide deletion appearing in some reads at the D614G position in the S protein was caused by the ARTIC Network tiled amplicon approach.

Investigation of the aligned reads around residue position 950 in the S protein revealed that inefficient primer trimming in Geneious lead to the alignment of an ARTIC Network V3 primer (nCoV-2019_81_LEFT), thereby masking the D950N mutation (Fig. 3C). We analysed over 1000 of our sequences defined as Delta and found the average frequency of the nucleotide A in the aligned reads (leading to the amino acid substitution D950N) to be 0.37 ± 0.16 (mean ± standard deviation). Again, this was far below the minimum variant frequency we set to call a base (0.70), which explains why this mutation was rarely detected in our S protein mutational analysis.

## Discussion

To prevent the spread of SARS-CoV-2 variants carrying novel mutations in the S protein, with significant implications for public health, early detection through whole-genome sequencing is imperative. In this way, timely preventive measures can be employed to reduce its spread. Here, we give one example, where a cluster of cases with a new SARS-CoV-2 variant (Delta + E484Q) was introduced into Uppsala County and was eventually eradicated by the time it was designated as a VUM by the ECDC. This was made possible by the early detection of this variant through genomic surveillance and the intensified control measures put forth by the local public health authorities, after it’s detection. These control measures included contact tracing, testing and isolation of all persons in direct contact with patients testing positive for Delta + E484Q [29]. Of note, variants expressing the E484Q mutation show reduced antibody neutralization against convalescent plasma [33]. This elegantly demonstrates the importance of rapid genomic surveillance combined with active public health measures at the local level. This can assist in preventing the spread of emerging variants with potentially enhanced infectivity or immune escape capabilities. Additionally, we demonstrate that genomic surveillance at the local level can give valuable information about the circulating variants nationally. Moreover, the distribution of VOC in Uppsala County matched remarkably well with the global pattern during 2021, which is not surprising considering the continuous influx of people into the county from other parts of Sweden and from abroad (https://www.epicov.org/). Consequently, we believe that Uppsala County is a representative cohort for genomic surveillance of SARS-CoV-2.

The Delta variant dominated Uppsala county over the summer and autumn seasons when SARS-CoV-2 transmission is reduced [34, 35]. We not only observed that the mutational frequency in the S protein of the Delta variant remained constant but also the cases were generally low throughout this period. Therefore, we speculate that if the Delta variant had been established in Uppsala County during the late autumn or winter seasons (or not been outcompeted by the Omicron variant in the early winter), the mutational frequency could have been higher, and we would have detected more novel mutations in the S protein. It should be noted that not a single S protein mutation we detected in our Delta sequences (apart from the signature mutations) became fixed in the virus population over time (that is, the mutation was detected in 100% of the SARS-CoV-2 sequences). It is possible that due to few cases/reduced spread of the Delta variant during summer and autumn, and also possibly due to the intensified control measures put forth by the local public health authorities in Uppsala County, random and beneficial mutations might have drifted out of the virus population by chance, as postulated by evolutionary dynamics [36]. Nevertheless, detection of transient S protein mutations can still prove valuable for predicting signature mutations or mutational sites in future emerging variants. We demonstrate this by the detection of two signature mutations for the Omicron variant in our Delta sequences, months before Omicron emerged. Moreover, we also detected four amino acid substitutions in the same residue position as Omicron signature mutations are located. Consequently, we have proposed six S protein mutations detected frequently in our Delta sequences throughout 2021 that should be monitored closely in the future (Table 1). It will be interesting to follow whether any of these mutations will play a role in shaping future SARS-CoV-2 variants.

By analysing the S protein mutations in 1053 Delta sequences sequenced by us, we observed that four Delta signature mutations were detected infrequently or not at all. Of note, three of these four Delta signature mutations were absent in 5–8% of all Delta sequences deposited globally, suggesting that this is a problem faced by laboratories sequencing SARS-CoV-2 genomes globally. Further investigation revealed that the source of these errors was from (1) incorrect amino acid mutation calling by the COVID-19 genome annotator tool and (2) systematic errors caused by the ARTIC Network SARS-CoV-2 tiled amplicon approach. The Delta variant’s signature triple mutation, E156/F157/R158G, was correctly called in our consensus nucleotide sequences, however, the COVID-19 genome annotator tool was unable to detect this non-codon-aligned triple mutation correctly. Moreover, uploading our S protein consensus nucleotide sequences to another online SARS-CoV-2 variant calling tool, named CoV-GLUE, yielded a similarly incorrect result [37]. We, therefore, speculate that a common pitfall of bioinformatics tools available for calling amino acid mutations from a SARS-CoV-2 nucleotide sequence is its inability to discern non-codon-aligned mutations/deletions. So, one should utilize these tools with caution, as important mutations might be incorrectly called/missed. However, it remains uncertain whether the GISAID database suffers from this error, considering ~ 8% of all the Delta sequences deposited globally lacked the E156/F157/R158G mutations.

One of the Delta variant’s signature mutations, D614G, was not detected in our Delta sequences, since a proportion of reads exhibited a frameshift deletion at this position. However, when using the Midnight protocol (utilizing a different primer scheme and slightly different library preparation steps) the D614G mutation was detected. We, therefore, suspect that the false frameshift deletion at this position might be due to the ARTIC Network tiled amplicon approach. On the other hand, a weakness of the Oxford Nanopore sequencing technology is its inability to correctly call bases in homopolymer tracts [38, 39]. Moreover, GC-rich homopolymer tracts of more than three bases are more prone to incorrect base-calling and commonly lead to a false nucleotide deletion [38, 40]. This matches our observation that the nucleotides GGG in the region surrounding D614G (in the reference genome: CAG GAT GTT; in the Delta variant: CAG GGT GTT) exhibit GG- (CAG G-T GTT) in some reads. So, this could also be a contributing factor to the low detection of the D614G mutation. The D614G mutation appeared during the sequencing runs when we lowered the barcode alignment score. This increases the overall output of classified reads at the cost of more misclassified reads with poor barcode sequence quality. During these runs, we also observed that more than 1,000 reads mapped to the SARS-CoV-2 reference sequence in our negative control (H_2_O), so this is not a recommended solution (data not shown).

D950N is another signature mutation of the Delta variant that was also detected at low levels in our Delta sequences. Because this position is part of a primer binding site for one of the ARTIC Network V3 primers, we suspect that inefficient primer trimming during assembly might have led to the masking of this mutation by the primer sequence. Considering that ~ 5% of all the Delta sequences deposited around the globe to the GISAID database are lacking this mutation, we speculate that this might be a sequencing error experienced by other users of the ARTIC Network SARS-CoV-2 tiled amplicon approach. However, this problem can be mitigated by using ARTIC Network improved V4 primers, where primers covering key mutational sites have been replaced [41].

## Conclusion

We report here the dynamics of SARS-CoV-2 VOC in a Swedish cohort during 2021. Our data matched the national VOC distribution during 2021, underscoring that genomic surveillance in a small, representative cohort can give valuable information about the dynamics at the national level. Further, we detected mutations attributable to VUM, VOI and future VOC (e.g., Omicron) in our Delta sequences. We also identified some less well-described S protein mutations in our Delta sequences that might play a role in future emerging variants. Additionally, multiple online SARS-CoV-2 variant calling tools were unable to recognize non-codon-aligned triple mutation and we recommend double-checking manually the results provided by such tools with the consensus sequences that one submitted before coming to any conclusion(s). Lastly, we report here multiple systematic errors that occurred when following the ARTIC Network SARS-CoV-2 tiled amplicon approach using the V3 primers and Nanopore sequencing, which led to the masking of some of the important signature mutations in the Delta sequences. This emphasizes the importance of not relying on only one approach for sequencing SARS-CoV-2 globally, as important emerging mutations during a pandemic could be missed. As the pandemic continues, sustained global sequencing efforts remain imperative to detect new variants early and to detect transient mutations in currently circulating variants, as they can give valuable clues to signature mutations of future VOC.

## Electronic supplementary material

Below is the link to the electronic supplementary material.

Supplementary Material 1

## Data Availability

The datasets generated and/or analysed during the current study are available in the GISAID repository, available at https://www.gisaid.org. The submission ID for all sequences is provided as an Excel file (see Supplementary Materials).

## Abbreviations

ACE2: angiotensin-converting enzyme 2.
ECDC: European Centre for Disease Prevention and Control.
GISAID: Global Initiative on Sharing Avian Influenza Data.
mAbs: monoclonal antibodies.
RBD: receptor-binding domain.
RdRp: RNA-dependent RNA polymerase.
RT-qPCR: quantitative reverse transcription PCR.
S: Spike.
VOC: variants of concern.
VOI: variants of interest.
VUM: variants under monitoring.
WHO: World Health Organization.
